# (*E*)-*N*′-[1-(2-Hydroxy­phen­yl)ethyl­idene]-3-methoxy­benzohydrazide

**DOI:** 10.1107/S1600536809010228

**Published:** 2009-03-28

**Authors:** Cong-Ming Li, Hong-Yan Ban

**Affiliations:** a College of Science, Shenyang University, Shenyang 110044, People’s Republic of China; b School of Chemical Engineering, University of Science and Technology Liaoning, Anshan 114051, People’s Republic of China

## Abstract

In the title compound, C_16_H_16_N_2_O_3_, the benzohydrazide group is not planar and the mol­ecule exists in a *trans* configuration with respect to the methyl­idene unit. The dihedral angle between the two substituted benzene rings is 26.9 (2)°. In the crystal structure, the mol­ecular packing is stabilized by intra­molecular O—H⋯N and inter­molecular N—H⋯O hydrogen bonds. The inter­molecular hydrogen bonding forms chains parallel to the *b* axis.

## Related literature

For the biological activities of hydrazones, see: Zhong *et al.* (2007 ▶); Raj *et al.* (2007 ▶); Jimenez-Pulido *et al.* (2008 ▶). For related structures, see: Ban & Li (2008*a* ▶,*b* ▶); Yehye *et al.* (2008 ▶); Fun *et al.* (2008*a* ▶,*b* ▶); Yang *et al.* (2008 ▶); Ejsmont *et al.* (2008 ▶).
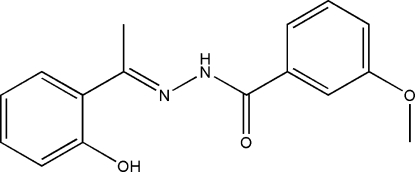


## Experimental

### 

#### Crystal data

C_16_H_16_N_2_O_3_
*M* *_r_* = 284.31
Orthorhombic, 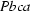

*a* = 12.932 (2) Å
*b* = 8.756 (2) Å
*c* = 25.784 (3) Å
*V* = 2919.7 (9) Å^3^
*Z* = 8
Mo *K*α radiation
μ = 0.09 mm^−1^
*T* = 298 K
0.27 × 0.23 × 0.20 mm

#### Data collection

Bruker SMART CCD area-detector diffractometer
Absorption correction: multi-scan (*SADABS*; Sheldrick, 1996 ▶) *T*_min_ = 0.976, *T*_max_ = 0.982
22735 measured reflections
3180 independent reflections
2023 reflections with *I* > 2σ(*I*)
*R*_int_ = 0.060

#### Refinement

*R*[*F* ^2^ > 2σ(*F* ^2^)] = 0.057
*wR*(*F* ^2^) = 0.145
*S* = 1.01
3180 reflections
196 parameters
1 restraint
H atoms treated by a mixture of independent and constrained refinement
Δρ_max_ = 0.17 e Å^−3^
Δρ_min_ = −0.13 e Å^−3^

### 

Data collection: *SMART* (Bruker, 1998 ▶); cell refinement: *SAINT* (Bruker, 1998 ▶); data reduction: *SAINT*; program(s) used to solve structure: *SHELXS97* (Sheldrick, 2008 ▶); program(s) used to refine structure: *SHELXL97* (Sheldrick, 2008 ▶); molecular graphics: *SHELXTL* (Sheldrick, 2008 ▶); software used to prepare material for publication: *SHELXTL*.

## Supplementary Material

Crystal structure: contains datablocks global, I. DOI: 10.1107/S1600536809010228/bx2198sup1.cif

Structure factors: contains datablocks I. DOI: 10.1107/S1600536809010228/bx2198Isup2.hkl

Additional supplementary materials:  crystallographic information; 3D view; checkCIF report

## Figures and Tables

**Table 1:** Hydrogen-bond geometry (Å, °)

*D*—H⋯*A*	*D*—H	H⋯*A*	*D*⋯*A*	*D*—H⋯*A*
N2—H2⋯O2^i^	0.897 (10)	2.010 (11)	2.894 (2)	168 (2)
O1—H1⋯N1	0.82	1.82	2.534 (2)	145

Symmetry code: (i) 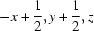.

**Fig. 1.**
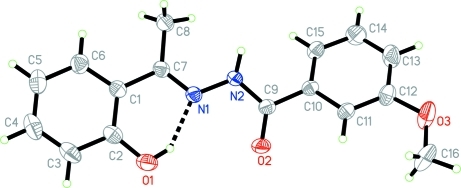
The molecular structure of (I), showing 30% probability displacement ellipsoids for the non-hydrogen atoms. Intramolecular O—H···N is shown as a dashed line.

**Fig. 2.**
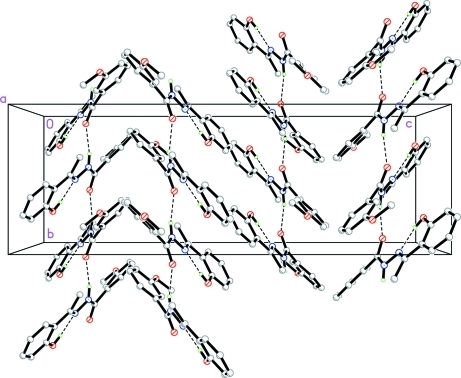
The packing diagram of (I), viewed along the a axis. Hydrogen bonds are shown as dashed lines.

## supplementary crystallographic information

### Comment

Hydrazones derived from the condensation of aldehydes with hydrazides have been demonstrated to possess excellent biological activities (Zhong *et al.*, 2007; Raj *et al.*, 2007; Jimenez-Pulido *et al.*, 2008). Due to the easy synthesis of such compounds, a great deal of hydrazones have been synthesized and structurally characterized (Yehye *et al.*, 2008; Fun *et al.*, 2008a,b; Yang *et al.*, 2008; Ejsmont *et al.*, 2008). Recently, we have reported two hydrazones (Ban & Li, 2008a,b). In this paper, we report herein the crystal structure of the title compound, (I). In the structure of (I), Fig. 1, the molecule exists in a *trans* configuration with respect to the methylidene unit. The dihedral angle between the two substituted benzene rings is 26.9 (2)°. In the 3-methoxyphenyl unit, the methoxy group is nearly coplanar with the mean plane of the C10–C15 ring, with the C16 atom deviates from the plane by 0.024 (2) Å. The torsion angle of C7-N1-N2-C9 is 8.0 (3)°. In the crystal structure the molecular packing is stabilized by intramolecular O-H···N and intermolecular N—H···O hydrogen bonds, Table 1. The intermolecular hydrogen bond form chains parallel to the *b* axis, Fig. 2.

### Experimental

The compound was prepared by refluxing 1-(2-hydroxyphenyl)ethanone (1.0 mol, 0136 g ) with 3-methoxybenzohydrazide (1.0 mol), 0166 g) in methanol (100 ml). Excess methanol was removed from the mixture by distillation. The colorless solid product was filtered, and washed three times with methanol. Colorless block crystals of the title compound were obtained from a methanol solution by slow evaporation in air.

### Refinement

H2 was located in a difference Fourier map and refined isotropically, with N–H distance restrained to 0.90 (1) Å. Other H atoms were placed in calculated positions (C–H = 0.93 - 0.96 Å, O–H = 0.82 Å) and refined as riding with U_iso_(H) = 1.2U_eq_(C) and 1.5U_eq_(O and methyl C). A rotating group model was used for the methyl groups.

### Crystal data

**Table:**

C_16_H_16_N_2_O_3_	*F*(000) = 1200
*M**_r_* = 284.31	*D*_x_ = 1.294 Mg m^−^^3^
Orthorhombic, *P**b**c**a*	Mo *K*α radiation, λ = 0.71073 Å
Hall symbol: -P 2ac 2ab	Cell parameters from 2030 reflections
*a* = 12.932 (2) Å	θ = 2.3–24.6°
*b* = 8.756 (2) Å	µ = 0.09 mm^−^^1^
*c* = 25.784 (3) Å	*T* = 298 K
*V* = 2919.7 (9) Å^3^	Block, colourless
*Z* = 8	0.27 × 0.23 × 0.20 mm

### Data collection

**Table:**

Bruker SMART CCD area-detector diffractometer	3180 independent reflections
Radiation source: fine-focus sealed tube	2023 reflections with *I* > 2σ(*I*)
graphite	*R*_int_ = 0.060
ω scans	θ_max_ = 27.0°, θ_min_ = 1.6°
Absorption correction: multi-scan (*SADABS*; Sheldrick, 1996)	*h* = −16→16
*T*_min_ = 0.976, *T*_max_ = 0.982	*k* = −11→11
22735 measured reflections	*l* = −32→32

### Refinement

**Table:**

Refinement on *F*^2^	Primary atom site location: structure-invariant direct methods
Least-squares matrix: full	Secondary atom site location: difference Fourier map
*R*[*F*^2^ > 2σ(*F*^2^)] = 0.057	Hydrogen site location: inferred from neighbouring sites
*wR*(*F*^2^) = 0.145	H atoms treated by a mixture of independent and constrained refinement
*S* = 1.01	*w* = 1/[σ^2^(*F*_o_^2^) + (0.0537*P*)^2^ + 0.9645*P*] where *P* = (*F*_o_^2^ + 2*F*_c_^2^)/3
3180 reflections	(Δ/σ)_max_ < 0.001
196 parameters	Δρ_max_ = 0.17 e Å^−^^3^
1 restraint	Δρ_min_ = −0.13 e Å^−^^3^

### Special details

**Table:**

Geometry. All esds (except the esd in the dihedral angle between two l.s. planes) are estimated using the full covariance matrix. The cell esds are taken into account individually in the estimation of esds in distances, angles and torsion angles; correlations between esds in cell parameters are only used when they are defined by crystal symmetry. An approximate (isotropic) treatment of cell esds is used for estimating esds involving l.s. planes.
Refinement. Refinement of F^2^ against ALL reflections. The weighted R-factor wR and goodness of fit S are based on F^2^, conventional R-factors R are based on F, with F set to zero for negative F^2^. The threshold expression of F^2^ > 2sigma(F^2^) is used only for calculating R-factors(gt) etc. and is not relevant to the choice of reflections for refinement. R-factors based on F^2^ are statistically about twice as large as those based on F, and R- factors based on ALL data will be even larger.

### Fractional atomic coordinates and isotropic or equivalent isotropic displacement parameters (Å^2^)

**Table:**

	*x*	*y*	*z*	*U*_iso_*/*U*_eq_	
O1	0.36951 (13)	−0.23812 (18)	0.55231 (7)	0.0649 (5)	
H1	0.3402	−0.1716	0.5692	0.097*	
O2	0.16810 (12)	−0.08166 (16)	0.63341 (7)	0.0637 (5)	
O3	−0.13883 (13)	0.2606 (3)	0.68697 (8)	0.0904 (7)	
N1	0.35910 (13)	−0.00001 (18)	0.60790 (6)	0.0449 (4)	
N2	0.29750 (13)	0.09206 (19)	0.63821 (7)	0.0455 (4)	
C1	0.51395 (16)	−0.0694 (2)	0.56716 (8)	0.0464 (5)	
C2	0.46801 (19)	−0.1972 (2)	0.54406 (8)	0.0531 (6)	
C3	0.5262 (2)	−0.2881 (3)	0.51064 (10)	0.0698 (7)	
H3	0.4956	−0.3729	0.4953	0.084*	
C4	0.6267 (3)	−0.2557 (3)	0.49994 (11)	0.0783 (9)	
H4	0.6643	−0.3189	0.4779	0.094*	
C5	0.6727 (2)	−0.1300 (3)	0.52166 (10)	0.0726 (8)	
H5	0.7413	−0.1067	0.5141	0.087*	
C6	0.61702 (17)	−0.0388 (3)	0.55467 (9)	0.0605 (6)	
H6	0.6489	0.0462	0.5692	0.073*	
C7	0.45502 (16)	0.0315 (2)	0.60247 (8)	0.0460 (5)	
C8	0.50836 (18)	0.1605 (3)	0.62968 (11)	0.0736 (8)	
H8A	0.5115	0.2475	0.6071	0.110*	
H8B	0.5772	0.1299	0.6389	0.110*	
H8C	0.4706	0.1867	0.6605	0.110*	
C9	0.20029 (16)	0.0434 (2)	0.64786 (8)	0.0453 (5)	
C10	0.13506 (15)	0.1510 (2)	0.67804 (7)	0.0416 (5)	
C11	0.02940 (15)	0.1511 (2)	0.66810 (8)	0.0472 (5)	
H11	0.0020	0.0846	0.6436	0.057*	
C12	−0.03443 (18)	0.2490 (3)	0.69451 (9)	0.0587 (6)	
C13	0.0058 (2)	0.3454 (3)	0.73150 (10)	0.0744 (8)	
H13	−0.0375	0.4117	0.7494	0.089*	
C14	0.1094 (2)	0.3437 (3)	0.74187 (10)	0.0741 (8)	
H14	0.1361	0.4087	0.7671	0.089*	
C15	0.17528 (17)	0.2464 (3)	0.71537 (9)	0.0551 (6)	
H15	0.2457	0.2454	0.7227	0.066*	
C16	−0.1850 (2)	0.1657 (4)	0.64939 (12)	0.0932 (10)	
H16A	−0.1543	0.1853	0.6162	0.140*	
H16B	−0.2578	0.1865	0.6478	0.140*	
H16C	−0.1743	0.0607	0.6586	0.140*	
H2	0.3118 (19)	0.1919 (13)	0.6414 (10)	0.080*	

### Atomic displacement parameters (Å^2^)

**Table:**

	*U*^11^	*U*^22^	*U*^33^	*U*^12^	*U*^13^	*U*^23^
O1	0.0719 (12)	0.0497 (10)	0.0731 (12)	−0.0087 (9)	−0.0043 (9)	−0.0107 (8)
O2	0.0519 (9)	0.0352 (8)	0.1039 (13)	−0.0034 (7)	−0.0095 (9)	−0.0069 (8)
O3	0.0454 (10)	0.1393 (19)	0.0865 (14)	0.0128 (11)	0.0099 (10)	−0.0058 (13)
N1	0.0432 (10)	0.0389 (9)	0.0524 (10)	0.0035 (8)	−0.0032 (8)	−0.0017 (8)
N2	0.0426 (10)	0.0339 (9)	0.0602 (11)	0.0017 (8)	0.0002 (8)	−0.0049 (9)
C1	0.0512 (13)	0.0411 (12)	0.0468 (12)	0.0051 (10)	−0.0031 (10)	0.0027 (9)
C2	0.0687 (16)	0.0415 (13)	0.0490 (13)	0.0059 (11)	−0.0025 (11)	0.0042 (10)
C3	0.104 (2)	0.0451 (15)	0.0607 (16)	0.0093 (14)	0.0061 (15)	−0.0058 (12)
C4	0.101 (2)	0.0675 (18)	0.0664 (17)	0.0287 (17)	0.0235 (16)	0.0042 (14)
C5	0.0695 (17)	0.0764 (19)	0.0720 (17)	0.0163 (15)	0.0202 (14)	0.0064 (15)
C6	0.0561 (15)	0.0587 (15)	0.0666 (15)	0.0054 (12)	0.0031 (12)	0.0024 (12)
C7	0.0440 (12)	0.0407 (11)	0.0534 (13)	0.0070 (9)	−0.0078 (10)	−0.0012 (10)
C8	0.0466 (14)	0.0697 (17)	0.105 (2)	0.0040 (12)	−0.0096 (14)	−0.0346 (16)
C9	0.0429 (12)	0.0363 (11)	0.0567 (13)	0.0008 (9)	−0.0096 (10)	0.0052 (10)
C10	0.0436 (11)	0.0387 (11)	0.0427 (11)	−0.0043 (9)	−0.0015 (9)	0.0073 (9)
C11	0.0445 (12)	0.0505 (13)	0.0466 (12)	−0.0060 (10)	0.0016 (10)	0.0068 (10)
C12	0.0467 (14)	0.0763 (17)	0.0531 (14)	0.0014 (12)	0.0097 (11)	0.0084 (13)
C13	0.0687 (18)	0.094 (2)	0.0608 (16)	0.0095 (15)	0.0184 (14)	−0.0177 (15)
C14	0.0771 (19)	0.092 (2)	0.0529 (15)	−0.0094 (16)	0.0051 (13)	−0.0249 (14)
C15	0.0464 (12)	0.0690 (15)	0.0498 (13)	−0.0057 (11)	−0.0005 (10)	−0.0029 (12)
C16	0.0430 (15)	0.140 (3)	0.097 (2)	−0.0071 (16)	−0.0068 (15)	0.012 (2)

### Geometric parameters (Å, °)

**Table:**

O1—C2	1.340 (3)	C6—H6	0.9300
O1—H1	0.8200	C7—C8	1.498 (3)
O2—C9	1.229 (2)	C8—H8A	0.9600
O3—C12	1.368 (3)	C8—H8B	0.9600
O3—C16	1.409 (4)	C8—H8C	0.9600
N1—C7	1.278 (2)	C9—C10	1.485 (3)
N1—N2	1.377 (2)	C10—C15	1.376 (3)
N2—C9	1.351 (3)	C10—C11	1.390 (3)
N2—H2	0.897 (10)	C11—C12	1.371 (3)
C1—C6	1.397 (3)	C11—H11	0.9300
C1—C2	1.401 (3)	C12—C13	1.376 (3)
C1—C7	1.480 (3)	C13—C14	1.367 (4)
C2—C3	1.394 (3)	C13—H13	0.9300
C3—C4	1.358 (4)	C14—C15	1.385 (3)
C3—H3	0.9300	C14—H14	0.9300
C4—C5	1.371 (4)	C15—H15	0.9300
C4—H4	0.9300	C16—H16A	0.9600
C5—C6	1.372 (3)	C16—H16B	0.9600
C5—H5	0.9300	C16—H16C	0.9600
			
C2—O1—H1	109.5	C7—C8—H8C	109.5
C12—O3—C16	118.2 (2)	H8A—C8—H8C	109.5
C7—N1—N2	119.81 (17)	H8B—C8—H8C	109.5
C9—N2—N1	117.28 (17)	O2—C9—N2	122.7 (2)
C9—N2—H2	118.8 (16)	O2—C9—C10	122.1 (2)
N1—N2—H2	120.2 (17)	N2—C9—C10	115.14 (18)
C6—C1—C2	117.4 (2)	C15—C10—C11	120.0 (2)
C6—C1—C7	121.3 (2)	C15—C10—C9	122.46 (19)
C2—C1—C7	121.4 (2)	C11—C10—C9	117.50 (18)
O1—C2—C3	117.3 (2)	C12—C11—C10	120.0 (2)
O1—C2—C1	123.3 (2)	C12—C11—H11	120.0
C3—C2—C1	119.3 (2)	C10—C11—H11	120.0
C4—C3—C2	121.6 (3)	O3—C12—C11	124.7 (2)
C4—C3—H3	119.2	O3—C12—C13	115.2 (2)
C2—C3—H3	119.2	C11—C12—C13	120.0 (2)
C3—C4—C5	120.0 (3)	C14—C13—C12	120.0 (2)
C3—C4—H4	120.0	C14—C13—H13	120.0
C5—C4—H4	120.0	C12—C13—H13	120.0
C4—C5—C6	119.5 (3)	C13—C14—C15	120.9 (2)
C4—C5—H5	120.2	C13—C14—H14	119.6
C6—C5—H5	120.2	C15—C14—H14	119.6
C5—C6—C1	122.2 (2)	C10—C15—C14	119.1 (2)
C5—C6—H6	118.9	C10—C15—H15	120.5
C1—C6—H6	118.9	C14—C15—H15	120.5
N1—C7—C1	115.99 (19)	O3—C16—H16A	109.5
N1—C7—C8	123.94 (19)	O3—C16—H16B	109.5
C1—C7—C8	120.07 (19)	H16A—C16—H16B	109.5
C7—C8—H8A	109.5	O3—C16—H16C	109.5
C7—C8—H8B	109.5	H16A—C16—H16C	109.5
H8A—C8—H8B	109.5	H16B—C16—H16C	109.5

### Hydrogen-bond geometry (Å, °)

**Table:**

*D*—H···*A*	*D*—H	H···*A*	*D*···*A*	*D*—H···*A*
N2—H2···O2^i^	0.90 (1)	2.01 (1)	2.894 (2)	168 (2)
O1—H1···N1	0.82	1.82	2.534 (2)	145

Symmetry codes: (i) −*x*+1/2, *y*+1/2, *z*.
